# Obesity, acute kidney injury and mortality in patients with sepsis: a cohort analysis

**DOI:** 10.1080/0886022X.2018.1430588

**Published:** 2018-02-01

**Authors:** Joana Gameiro, Miguel Gonçalves, Marta Pereira, Natacha Rodrigues, Iolanda Godinho, Marta Neves, João Gouveia, Zélia Costa e Silva, Sofia Jorge, José António Lopes

**Affiliations:** a Division of Nephrology and Renal Transplantation, Department of Medicine, Centro Hospitalar Lisboa Norte, EPE, Lisboa, Portugal;; b Division of Intensive Medicine, Department of Medicine, Centro Hospitalar Lisboa Norte, EPE, Lisboa, Portugal

**Keywords:** Obesity, acute kidney injury, epidemiology, intensive care, sepsis, survival analysis

## Abstract

Although the prognostic effect of obesity has been studied in critically ill patients its impact on outcomes of septic patients and its role as a risk factor for acute kidney injury (AKI) is not consensual. We aimed to analyze the impact of obesity on the occurrence of AKI and on in-hospital mortality in a cohort of critically ill septic patients. This study is retrospective including 456 adult patients with sepsis admitted to the Division of Intensive Medicine of the Centro Hospitalar Lisboa Norte (Lisbon, Portugal) between January 2008 and December 2014. Obesity was defined as a body mass index of 30 kg/m^2^ or higher. The Kidney Disease Improving Global Outcomes classification was used to diagnose and classify patients developing AKI. AKI occurred in 87.5% of patients (19.5% with stage 1, 22.6% with stage 2 and 45.4% with stage 3). Obese patients developed AKI more frequently than non-obese patients (92.8% versus 85.5%, *p* = .035; unadjusted OR 2.2 (95% CI: 1.04–4.6), *p* = .039; adjusted OR 2.31 (95% CI: 1.07–5.02), *p* = .034). The percentage of obese patients, however, did not differ between AKI stages (stage 1, 25.1%; stage 2, 28.6%; stage 3, 15.4%; *p* = .145). There was no association between obesity and mortality (*p* = .739). Of note, when comparing AKI patients with or without obesity in terms of in-hospital mortality there were also no significant differences between those groups (38.4% versus 38.4%, *p* = .998). Obesity was associated with the occurrence of AKI in critically ill patients with sepsis; however, it was not associated with in-hospital mortality.

## Introduction

Obesity is considered as a major public health problem and has been shown to be associated with an increased all-cause mortality, cardiovascular disease (CVD) and diabetes mellitus [1]. The high prevalence of obesity in the general population has led to a higher number of obese patients being hospitalized in the intensive care unit (ICU). Although the prognostic effect of obesity has been extensively studied in critically ill patients [2,3], the impact of obesity on outcomes of patients with sepsis is not well studied. In fact, the relationship between obesity and sepsis mortality is inconsistent in the literature [1,4,5].

Obesity has also been associated with chronic kidney disease (CKD) [6,7], however, its role as a risk factor for acute kidney injury (AKI) is still not consensual [8,9].

The foremost cause of AKI in the ICU is sepsis and patients with non-septic AKI are clinically different from those suffering from AKI of septic origin. In actual fact, septic AKI is associated with higher disease severity scores at admission into the ICU, requirement of vasoactive drugs, need for mechanical ventilation, non-renal organ failure, prolonged lengths of ICU and hospital stay, increased in-hospital mortality and a higher probability of recovery of renal function at the time of discharge from hospital [10,11]. Subsequently, the need for a profound understanding and purview of septic AKI by the nephrologist and the intensivist is indispensable as a means to ensure adequate diagnoses, treatment decisions, follow-up strategies and, ultimately, predicting patient outcome.

The aim of this study was to analyze the impact of obesity on the occurrence of AKI and on in-hospital mortality in a cohort of critically ill patients admitted with sepsis. For this purpose, we cross-examined data from a retrospective study in which we studied a cohort of critically ill patients admitted with sepsis to the ICU in which the objective was to compare the diagnostic sensitivity and prognostic ability of the standard classifications for AKI, namely the ‘Risk, Injury, Failure, Loss of kidney function, End-stage kidney disease’, Acute Kidney Injury Network and Kidney Disease: Improving Global Outcomes (KDIGO) classifications [12]. We performed a similar analysis from this data to study the association of serum lactates and AKI [13].

## Materials and methods

This is a retrospective study including all patients with sepsis admitted to the Division of Intensive Medicine of the Centro Hospitalar Lisboa Norte (Lisbon, Portugal), an academic and referral center covering 3,000,000 inhabitants, between January 2008 and December 2014. The study was approved by the Ethical Committee at the Centro Hospitalar Lisboa Norte, EPE, in agreement with institutional guidelines. Informed consent was waived by the Ethical Committee due to the retrospective and non-interventional nature of the study.

### Participants

Eligible patients were selected according to the ICU patient admission register as adult patients (≥18 years of age) with a diagnosis of sepsis or septic shock at admission to the Division of Intensive Medicine. The third international consensus definitions for sepsis and septic shock were used [14]. Exclusion criteria were defined and comprised (i) CKD patients on renal replacement therapy, (ii) patients who underwent renal replacement therapy 1 week prior to admission to the ICU and (iii) patients who were discharged or died less than 2 d after ICU admission.

### Variables and data sources

All patient variables were collected from individual clinical records. We analyzed the following variables: patient demographic characteristics (age, gender, ethnicity, body weight and height), comorbidities (presence of diabetes mellitus, hypertension, chronic obstructive pulmonary disease (COPD), CVD, cirrhosis and/or malignancy), primary diagnosis on admission (medical versus surgical in nature), source of infection, serum hemoglobin, serum albumin, serum creatinine (SCr), urine output (UO), disease severity according to the Simplified Acute Physiologic Score (SAPS) II [15] as determined by the worst variables recorded during the first 24 h, fluid balance, mechanical ventilation, vasopressor use and requirement for renal replacement therapy. Diabetes mellitus was diagnosed according to the American Diabetes Association criteria [16] and hypertension was diagnosed according to the seventh report of the Joint National Committee [17]. COPD included emphysema and chronic bronchitis and CVD was considered as present whenever a history of cerebrovascular disease, chronic heart failure of any cause, cardiac ischemic disease and/or peripheral arterial disease was documented, also, a previous diagnosis on clinical records was considered sufficient for the confirmation of this diagnosis. The outcomes measured were AKI and in-hospital mortality.

Obesity was defined as a body mass index (BMI) of 30 kg/m^2^ or higher, and underweight was defined as a BMI lower than 18.5 kg/m^2^ [18].

AKI was diagnosed within the first week of ICU hospitalization based on the KDIGO classification according to both SCr and UO criteria (Table 1). In this ICU, the protocol for all patients includes daily determination of SCr and hourly UO. Pre-admission SCr (SCr within the previous 3 months) were considered as baseline values. When these values were unavailable, baseline SCr was estimated from the modification of diet in renal disease (MDRD) equation [19], accepting the lower limit of a normal baseline glomerular filtration rate (GFR) of 75 ml/min/1.73 m^2^.

**Table 1.** Kidney disease improving global outcomes (KDIGO) classification.

Stage	SCr/GFR	UO
1	↑ SCr ≥26.5 μmol/l (≥0.3 mg/dl) or ↑ SCr ≥150–200% (1.5–1.9X)	<0.5 ml/kg/h (>6 h)
2	↑ SCr >200–300% (>2–2.9X)	<0.5 ml/kg/h (>12 h)
3	↑ SCr >300% (≥3X) or ↑ SCr to ≥353.6 μmol/l (≥4 mg/dl) or initiation of renal replacement therapy	<0.3 ml/kg/h (24 h) or anuria (12 h)

### Statistical methods

Categorical variables were described as the total number and percentage for each category, whereas continuous variables were described as the mean ± standard deviation. Normally distributed continuous variables were compared with the Student’s *t*-test, non-normally distributed continuous variables were compared with the Mann–Whitney U test and categorical variables were compared with the chi-square test. To determine risk factors for AKI and for in-hospital mortality, univariate and multivariate logistic regression analysis was employed. In the multivariate analysis (enter model), only variables with statistical significance in the univariate analysis were included. Data were expressed as odds ratios (ORs) with 95% confidence intervals (CIs). Additionally, a sensitivity analysis excluding underweight patients was performed to eliminate the potential confound of this variable on the occurrence of AKI and in mortality. Statistical significance was defined as a *p* value <.05. Statistical analysis was performed with the statistical software package SPSS for Windows (version 21.0; SPSS, Chicago, IL).

## Results

### Participants

Of the 722 potentially eligible patients, 266 were excluded based on the following reasons: 122 had stage 5 CKD on renal replacement therapy and 144 had been hospitalized for less than 48 h. No patients required renal replacement therapy in the week preceding ICU admission. Accordingly, we studied a final cohort of 456 patients. Baseline characteristics are described in Table 2, while clinical and demographic characteristics of the studied patients according to AKI and obesity are shown in Tables 3 and 4.

**Table 2.** Patients’ baseline characteristics.

Characteristics	Value
Age (year)	64.1 ± 16.4
Gender (Male) – *n* (%)	264 (57.9)
Race (Caucasian) – *n* (%)	433 (94.7)
Obese (IMC> =30) – *n* (%)	125 (27.4)
Underweight (IMC <18.5) – *n* (%)	9 (2)
Hypertension – *n* (%)	212 (46.5)
Comorbidities – *n* (%)	
Diabetes	103 (22.6)
CVD	125 (27.4)
COPD	38 (8.3)
Cirrhosis	18 (3.9)
Neoplasia	109 (23.9)
Medical admission – *n* (%)	253 (55.5)
Infection source – *n* (%)	
Abdominal	187 (41.0)
Respiratory	138 (30.3)
Kidney	57 (12.5)
Skin	34 (7.5)
Others	26 (5.7)
Unknown	14 (3.1)
SAPS II	49.4 ± 17.3
Baseline SCr (mg/dl)	1.3 ± 0.6
Admission SCr (mg/dl)	2.3 ± 1.5
Hemoglobin (g/dl)	10.4 ± 2.0
Serum albumin (g/dl)	1.9 ± 0.6
Mechanical ventilation – *n* (%)	350 (76.8)
Vasopressors – *n* (%)	316 (369.3)
Fluid balance (l)	4.5 ± 5.7
RRT – *n* (%)	108 (23.7)
LOS in hospital (days)	37.1 ± 39.4
LOS in ICU (days)	10.0 ± 10.0
ICU mortality – *n* (%)	108 (23.7)
In-hospital mortality – *n* (%)	153 (33.6)

**Table 3.** Demographic and clinical characteristics of patients according to the development of acute kidney injury defined by the KDIGO classification.

	KDIGO		
Characteristics	No AKI (*n* = 57)	AKI (*n* = 399)	*p* Value
Age (year)	64.1 ± 17.4	64.1 ± 16.0	.994
Gender (Male) – *n* (%)	35 (61.4)	229 (57.4)	.566
Race (Caucasian) – *n* (%)	54 (94.7)	379 (95.0)	.936
Obesity – *n* (%)	9 (15.7)	116 (29.1)	.035
Underweight – *n* (%)	3 (5.3)	6 (1.5)	.242
Co-morbidities – *n* (%)			
Hypertension	30 (50.2)	182 (45.6)	.320
Diabetes	15 (26.3)	88 (22.1)	.472
CVD	14 (24.6)	111 (27.8)	.606
COPD	5 (8.8)	33 (8.3)	.898
Cirrhosis	1 (1.8)	17 (4.3)	.363
Neoplasia	13 (22.8)	96 (24.1)	.836
Medical admission – *n* (%)	31 (54.4)	222 (55.6)	.859
Infection source – *n* (%)			
Abdominal	19 (33.3)	168 (42.1)	.208
Respiratory	16 (28.3)	122 (30.6)	.700
Kidney	14 (24.6)	43 (10.8)	.003
Skin	2 (3.5)	32 (8.0)	.225
Others	6 (10.5)	20 (5.0)	.093
Unknown	0 (0.0)	14 (3.5)	.151
SAPS II	42.8 ± 15.6	50.4 ± 17.3	.002
Baseline SCr (mg/dl)	1.4 ± 0.7	1.3 ± 0.6	.185
Hemoglobin (g/dl)	10.0 ± 1.7	10.5 ± 2.0	.078
Serum albumin (g/dl)	2.1 ± 0.6	1.9 ± 0.6	.003
Mechanical ventilation – *n* (%)	41 (71.9)	309 (77.4)	.357
Vasopressors – *n* (%)	26 (45.6)	290 (72.7)	<.001
RRT – *n* (%)		108 (27.1)	
LOS in hospital (days)	39.5 ± 44.8	36.7 ± 38.6	.625
LOS in ICU (days)	9.8 ± 10.3	10.0 ± 10.0	.881
ICU mortality – *n* (%)	7 (12.3)	101 (25.3)	.030
Hospital mortality – *n* (%)	10 (17.5)	143 (87.5)	.006

**Table 4.** Demographic and clinical characteristics of patients according to obesity.

Characteristics	Non-obese patients (*n* = 331)	Obese patients (*n* = 125)	*p* Value
Age (year)	63.9 ± 16.5	64.4 ± 14.8	.779
Gender (Male) – *n* (%)	203 (61.3)	61 (48.8)	.016
Race (Caucasian) – *n* (%)	309 (93.4)	124 (99.2)	.011
AKI – *n* (%)	283 (85.5)	116 (92.8)	.035
Comorbidities – *n* (%)			
Hypertension	141 (43)	71 (56.8)	.007
Diabetes	63 (19)	40 (32)	.003
CVD	98 (29.6)	27 (21.6)	.087
COPD	32 (9.7)	6 (4.8)	.093
Cirrhosis	12 (3.6)	6 (4.8)	.566
Neoplasia	93 (28.1)	16 (12.8)	.001
Medical admission – *n* (%)	188 (56.8)	65 (52)	.398
Infection source – *n* (%)			
Abdominal	132 (56.8)	55 (44)	.425
Respiratory	111 (33.5)	27 (21.6)	.013
Kidney	37 (11.2)	20 (16)	.165
Skin	25 (7.6)	9 (7.2)	.898
Others	18 (5.4)	8 (6.4)	.693
Unknown	8 (2.4)	6 (4.8)	.188
SAPS II	48.4 ± 17.3	52.1 ± 17.1	.043
Baseline SCr (mg/dl)	1.3 ± 0.58	1.3 ± 0.67	.524
Hemoglobin (g/dl)	10.2 ± 2	10.8 ± 2	.005
Serum albumin (g/dl)	1.9 ± 0.6	1.9 ± 0.5	.896
Mechanical ventilation – *n* (%)	256 (77.3)	94 (75.2)	.629
Vasopressors – *n* (%)	228 (68.9)	88 (70.4)	.754
RRT – *n* (%)	64 (19.3)	44 (35.2)	<.001
LOS in hospital (days)	38.8 ± 39.3	32.6 ± 39.3	.136
LOS in ICU (days)	10 ± 9.8	10 ± 10.7	.91
ICU mortality – *n* (%)	81 (24.5)	27 (21.6)	.520
Hospital mortality – *n* (%)	113 (34.1)	40 (32)	.666

Obese patients were more likely to be female (*p* = .016) and Caucasian (*p* = .011), and, as expected, to have prior hypertension (*p* = .007) and diabetes (*p* = .003). Furthermore, neoplasia (*p* = .001) and respiratory origin (*p* = .013) were less frequent in obese patients. Obese patients had higher SAPS II values (*p* = .043) and hemoglobin (*p* = .005) at ICU admission.

Pre-admission SCr was available in 185 patients (40.6%) and in the remaining cases [*n* = 272 (59.4%)] estimation using the MDRD formula was necessary. AKI occurred in 87.5% of patients with a maximum KDIGO category (19.5% with stage 1, 22.6% with stage 2 and 45.4% with stage 3). One-hundred and eight patients (23.7%) underwent renal replacement therapy (8.3% intermittent hemodialysis, 76% HDFVVC, and 15.7% both). AKI developed more frequently in patients with urinary tract infections (*p* = .003), with higher SAPS II values (*p* = .002) and lower albumin values (*p* = .003). Also, AKI patients were more likely to require vasopressors (*p* < .001).

### Obesity and AKI

Obese patients developed AKI more frequently than non-obese patients (92.8% versus 85.5%, *p* = .035; unadjusted OR 2.2 (95% CI: 1.04–4.6), *p* = .039; adjusted OR 2.31 (95% CI: 1.07–5.02), *p* = .034). The percentage of obese patients, however, did not differ between AKI stages (stage 1, 25.1%; stage 2, 28.6%; stage 3, 15.4%; *p* = .145) (Tables 3, 4 and 6). After excluding underweight patients, obesity still remained associated with AKI (92.8% versus 85.1%, *p* = .028; unadjusted OR 2.2 (95% CI: 0.015–0.264), *p* = .028; adjusted OR 2.3 (95% CI: 0.011–0.146), *p* = .024).

**Table 6.** Univariate and multivariate analysis of factors predictive of outcomes.

	AKI				Mortality			
	Unadjusted HR (95% CI)	*p* Value	Adjusted HR (95% CI)	*p* Value	Unadjusted HR (95% CI)	*p* Value	Adjusted HR (95% CI)	*p* Value
Demographics								
** **Age	1.00 (0.98–1.02)	.094			1.02 (1.08–1.04)	.001	1.02 (1.00–1.03)	.026
** **Male	0.85 (0.48–1.50)	.567			1.20 (0.81–1.18)	.375		
** **Caucasian	1.053 (0.30–3.66)	.936			0.64 (0.27–1.50)	.304		
** **Obesity	2.19 (1.04–4.60)	.039	2.31 (1.07–5.02)	.034	0.91 (0.59–1.41)	.666		
** **Underweight	−1.2 (−0.29–0.07)	.243			0.38 (−0.2–0.31)	.704		
Comorbidities								
** **Hypertension	0.76 (0.43–1.32)	.321			0.88 (0.60–1.31)	.534		
** **Diabetes	0.79 (0.42–1.50)	.473			0.97 (0.61–1.54)	.894		
** **CVD	1.18 (0.62–2.25)	.606			0.91 (0.59–1.41)	.666		
** **COPD	0.94 (0.35–2.51)	.898			1.68 (0.86–3.29)	.131		
** **Cirrhosis	2.492 (0.33–19.09)	.379			1.27 (0.48–3.35)	.625		
** **Neoplasia	1.04 (0.75–1.44)	.836			1.42 (1.14–1.77)	.002	1.2 (0.93–1.55)	.166
** **Medical admission	1.05 (0.60–1.84)	.859			1.00 (0.68–1.49)	.982		
Infection source								
** **Abdominal	1.45 (0.81–2.61)	.210			1.18 (0.80–1.76)	.391		
** **Respiratory	1.13 (0.61–2.09)	.700			1.08 (0.71–1.65)	.714		
** **Kidney	0.37 (0.19–0.73)	.004	0.39 (0.19–0.82)	.013	0.38 (0.19–0.78)	.008	0.40 (0.17–0.93)	.034
** **Skin	2.40 (0.56–10.29)	.239			1.25 (0.61–2.56)	.548		
** **Others	0.45 (0.17–1.17	.101			1.05 (0.46–2.42)	.906		
SAPS II	1.03 (1.01–1.05)	.002	1.01 (0.99–1.03)	.187	1.05 (1.04–1.06)	.000	1.04 (1.02–1.06)	.000
Baseline SCr	0.76 (0.50–1.15)	.188			0.89 (0.64–1.23)	.469		
Hemoglobin	1.14 (0.99–1.32)	.078			0.80 (0.72–0.89)	.000	0.79 (0.70–0.89)	.000
Serum albumin	0.51 (0.33–0.81)	.004	0.67 (0.41–1.09)	.105	0.56 (0.39–0.81)	.002	0.91 (0.60–1.39)	.661
Mechanical ventilation	1.34 (0.72–2.50)	.358			3.07 (1.77–5.33)	.000	1.78 (0.94–3.40)	.079
Vasopressors	3.17 (1.80–5.59)	.000	2.63 (1.37–5.04)	.004	2.34 (1.47–3.71)	.000	1.1 (0.62–1.94)	.749
AKI					2.63 (1.29–5.35)	.008	2.44 (1.07–5.57)	.035

Obese patients developing AKI were more likely to require renal replacement therapy than non-obese AKI patients (37.9% versus 22.6%, *p* < .001).

### Obesity and in-hospital mortality

There was no association between obesity and mortality (*p* = .739) (Tables 4–6). After excluding underweight patients, the same findings were observed (32% versus 33.9%, *p* = .709; unadjusted OR −0.4 (95% CI: −0.12–0.08), *p* = .71). Of note, when comparing AKI patients with or without obesity in terms of in-hospital mortality there were also no significant differences between those groups (32% versus 34.1%, *p* = .666).

**Table 5.** Demographic and clinical characteristics of patients according to in-hospital mortality.

Characteristics	No in-hospital mortality 303	In-hospital mortality 153	*p* Value
Age (year)	62.32 ± 15.9	67.54 ± 15.8	.001
Gender (Male) – *n* (%)	171 (56.4)	93 (60.8)	.374
Race (Caucasian) – *n* (%)	290 (95.7)	143 (93.5)	.301
Obesity – *n* (%)	85 (28.1)	40 (26.1)	.739
Comorbidities – *n* (%)			
Hypertension	144 (47.5)	68 (44.4)	.533
Diabetes	69 (22.8)	34 (22.2)	.894
CVD	85 (28.1)	40 (26.1)	.666
COPD	21 (6.9)	17 (11.1)	.127
Cirrhosis	11 (3.6)	7 (4.6)	.625
Neoplasia	59 (19.5)	50 (32.7)	.002
Medical admission – *n* (%)	168 (55.4)	85 (55.6)	.982
Infection source – *n* (%)			
** **Abdominal	120 (39.6)	67 (43.8)	.391
Respiratory	90 (29.7)	48 (31.4)	.714
Kidney	47 (15.5)	10 (6.5)	.006
Skin	21 (5.9)	13 (8.5)	.574
Others	17 (5.6)	9 (6)	1.000
Unknown	8 (2.6)	6 (3.9)	.566
SAPS II	45.13 ± 15.4	57.94 ± 16.7	<.001
Baseline SCr (mg/dl)	1.3 ± 0.64	1.25 ± 0.54	.47
Hemoglobin (g/dl)	10.7 ± 2	9.9 ± 2	<.001
Serum albumin (g/dl)	1.98 ± 0.57	1.79 ± 0.6	.002
Mechanical ventilation – *n* (%)	215 (71)	135 (88.2)	<.001
Vasopressors – *n* (%)	193 (63.7)	123 (80.4)	<.001
RRT – *n* (%)	43 (14.2)	65 (42.5)	<.001
AKI – *n* (%)	256 (84.5)	143 (93.5)	.006
LOS in hospital (days)	37.97 ± 38	35.3 ± 41.9	.494
LOS in ICU (days)	9.6 ± 9.6	10.7 ± 10.9	.261

## Discussion

To our knowledge, this is the first study aiming to evaluate the association between obesity and AKI in septic patients and its impact in mortality. In this retrospective study including 456 critically ill patients with sepsis, we found that obesity was independently associated with AKI, increasing the risk for developing AKI.

Our results are in line with other studies which established an association between obesity and risk of AKI in critically ill patients or in post-operative patients. In a study by Danzinger et al., in a population of 14,986 critically ill patients admitted to a medical or surgical ICU, obese patients developed more AKI [20]. Thakar et al. noted a prevalence of AKI of 8.5% regarding 491 patients submitted to bariatric surgery and described its impact in prolonging the length of hospital stay [21]. Yap et al. found that obesity and morbid obesity were associated with an increased risk for AKI [22]. Billings et al. reported BMI as an independent risk factor for the development of AKI in a population of 112 patients after cardiac surgery [23]. Kumar et al. detailed an increased risk for AKI in patients with BMI > 40 kg/m^2^ in 376 patients who underwent cardiac surgery [24] Soto et al. demonstrated that an increasing BMI was significantly associated with development of AKI in a cohort of 715 patients with Acute Respiratory Distress Syndrome [25]. Plataki et al. demonstrated that BMI was independently associated with AKI in ICU patients with septic shock [26].

Obesity dramatically alters renal hemodynamics, which could explain an increased susceptibility to AKI in obese patients [27]. Although the pathophysiology is not completely understood, the increased renal plasma flow and GFR resulting from altered hemodynamics could lead to a higher filtration fraction or hyperfiltration syndrome, potentially contributing to an increased renal susceptibility to damage [28]. A recent study by Billings et al. found that markers of oxidative stress appear to affect renal function by reducing renal perfusion, reducing creatinine clearance, and instigating cellular and functional renal damage in experimental models, and demonstrated that these biomarkers were a strong predictor of AKI in cardiac surgery patients [23]. Adipose production of inflammatory mediators, as well as adipokines such as leptin, and decreased production of adiponectin, in response to acute illness have been associated with a heightened AKI risk [29]. Another factor that could contribute to obesity’s influence on AKI is the challenge to correctly assess the intravascular volume status and to prescribe adequate fluid therapy and/or vasopressors. Dosing of potentially nephrotoxic agents may also be demanding, since obesity impacts many pharmacokinetic factors, and the dose itself may be variable depending on whether it was based on actual body weight or on formulae-based weight adjustment, not obesity [30]. Notably, obese ICU patients are at an increased risk for elevated intra-abdominal pressure, which may, in itself, cause renal dysfunction from both venous congestion and poor arterial organ perfusion [31,32].

Cardiac changes associated with obesity, such as increased left ventricular hypertrophy and direct infiltration of the myocardium might also alter renal perfusion [33]. Consequently, it is plausible that kidney and/or cardiac-specific changes associated with obesity contribute to the prevalence of AKI.

Although not the scope of this study, our results also confirmed an association between AKI and mortality in septic patients (93.5% versus 84.5%, *p* = .006; unadjusted OR 2.63 (95% CI: 1.29–5.35), *p* = .008; adjusted OR 2.44 (95% CI: 1.07–5.57), *p* = .035), which is in accordance with previously published studies of AKI in the critically ill patient [34,35].

The impact of obesity on mortality in septic patients still remains unclear. Our results are in concordance with other studies that did not demonstrate any relationship between obesity and mortality in septic patients. Sakr et al. performed a sub-study based on the observational sepsis occurrence in acutely ill patients (SOAP) study, enrolling 198 ICUs in 24 European countries and did not find any statistically significant association between obesity and sepsis mortality [5]. Similar findings were described by Chalkias et al. in a small prospective cohort study [6]. Remarkably, in a study by Nguyen et al. obesity was associated with a significant decrease in mortality among hospitalized septic patients; nevertheless, it was also associated with greater duration and cost of hospitalization [36]. Prescott et al. also reported that obesity was independently associated with decreased mortality up to 1 year after hospitalization in an observational study of 1404 severe sepsis patients [37]. On contrary, Huttunen et al. reported a higher risk of death in obese patients with sepsis [38].

The term ‘obesity paradox’ has been used to describe similar or improved survival for obese critically ill patients notwithstanding concomitant comorbidities. One explanation proposed for this phenomenon is that obese patients benefit from a metabolic or nutritional reserve, which ultimately culminates in an increased survival in conditions of illness [39]. Another hypothesis is that there is an underlying difference in metabolic and immune responses to acute illness which may act as a counterbalance to excess inflammation [40,41]. However, another plausible justification is the presence of methodological issues observed in the aforementioned studies.

In the present study, some limitations have to be acknowledged. First, the single-center and retrospective nature of the study with a small cohort of patients may compromise, at least in part, the results of our study. Second, although hourly UO measurements were available to us for collection and analysis, data regarding additional factors that could potentially influence UO, such as the use of diuretic therapy, was not. Third, the pre-admission SCr level was unknown in nearly 60% of patients, compelling us to calculate an estimated baseline function using the MDRD equation, albeit as recommended (assuming the lower limit of the normal baseline GFR 75 ml/min/1.73 m^2^). In addition, it addresses only all-cause mortality and not cause-specific mortality or morbidity, and it also addresses only findings related to BMI and not to other aspects of body composition, such as visceral fat or fat distribution. Lastly, the pathophysiologic mechanisms on the association between obesity and AKI were not examined.

Despite these limitations, our study has several noteworthy strengths. To the best of our knowledge, this is the first study evaluating the association between obesity and AKI and between obesity and in-hospital mortality in septic patients in an ICU. Additionally, we used not only creatinine criteria but also UO criteria to diagnose and categorize AKI. Finally, despite the retrospective nature of the study, most of the studied variables were registered as part of routine clinical practice on a daily basis and made accessible for analysis.
